# Identifying safety zone of invasive procedures in the sternocleidomastoid muscle using ultrasonography

**DOI:** 10.1097/MD.0000000000033021

**Published:** 2023-02-22

**Authors:** Byung Heon Kang, So Hyun Park, Seok Kang, Joon Shik Yoon

**Affiliations:** a Department of Physical Medicine and Rehabilitation, Korea University Guro Hospital, Korea University College of Medicine, Seoul, Korea.

**Keywords:** great auricular nerve, safety zone, spinal accessory nerve, sternocleidomastoid muscle, transverse cervical nerve, ultrasonography

## Abstract

Dysfunctions of the sternocleidomastoid (SCM) muscle, such as myofascial syndrome, torticollis, and cervical dystonia, have been treated using several invasive procedures. In such situations, it is possible to injure the adjacent nerves. This study aimed to demonstrate the course of these nerves in healthy volunteers using ultrasound. The great auricular nerve (GAN), spinal accessory nerve (SAN), transverse cervical nerve (TCN), and supraclavicular nerve (SCN) were scanned by ultrasonography in 26 healthy volunteers. The neck was scanned in the supine position with the head turned 45° to the contralateral side. The cervical plexus was detected in half of the SCM muscle. Each nerve was then traced to the level of contact with the anterior border of the SCM muscle. The following features of the nerves were recorded bilaterally: vertical and horizontal positions of each nerve at the posterior border of the SCM and the cross-sectional area and depth of each nerve at the reference line and anterior border of the SCM. The mean proportions of GAN, SAN, TCN, and SCN were 26%, 26%, 48%, and 80%, respectively near the posterior border, whereas they were 18%, 23%, and 51% for GAN, SAN, and TCN, respectively, at the level of the reference line. Notably, SCN was not visible at the level of the reference line. The mean TCN proportion was 47% at the anterior border of the SCM. The precise location of the nerves and their relationship with the SCM muscle should be considered during invasive procedures. It is recommended that the procedure be performed in the lower half of the SCM muscle, which refers to 50 to 80% of the proportions in our study.

## 1. Introduction

The sternocleidomastoid (SCM) is one of the most superficial cervical muscles. This muscle originates from 2 locations which include the manubrium of the sternum and the clavicle.^[1]^ Cervical plexus is located deep in the SCM muscle,^[2]^ and it originates from the anterior rami of C1 to C4 spinal nerves. After forming a plexus, it gives branches (at the midway) at the posterior border of the SCM muscle, including the great auricular nerve (GAN), transverse cervical nerve (TCN), supraclavicular nerve (SCN), and muscular branches.^[3]^ Another nerve passing down the SCM is the spinal accessory nerve (SAN) which supplies the SCM and trapezius muscles. After the accessory nerve leaves the skull, the spinal component of the accessory nerve travels back and downwards, below which it pierces and supplies the motor to the SCM muscle. It then emerges at the posterior triangle of the neck and continues to innervate the upper part of the trapezius muscle.

Considerable numbers of nerves pass around the SCM muscle, and hence, injury to these nerves is most commonly caused by invasive procedures targeting the SCM muscle.^[4]^ In clinical practices, physicians inject mixtures of steroid and lidocaine, alcohol, and botulinum toxin to treat dysfunction of SCM involving myofascial syndrome, torticollis^[5]^, and cervical dystonia.^[6]^ Typically, in the patient with myofascial pain syndromes, physicians palpate the trigger point in SCM and perform the trigger point injection in all the areas in the SCM with blind technique.^[7]^ Meanwhile, physicians usually target the upper third of the SCM during botulinum neurotoxins injection to minimize botulinum toxin diffusion to the swallowing area.^[6]^

Injury to the cutaneous nerves might cause relatively small complications such as tingling sensation, numbness, and paresthesia, while damage to the motor branch might cause tragic neck and shoulder weakness, which might disable daily activity.^[8]^

Based on anatomical studies,^[3]^ vulnerable nerves are located mostly in the upper and middle thirds of the SCM. Therefore, we hypothesized that the lower third of the SCM might be a better location for minimizing iatrogenic nerve injury during the blind technique. This study aimed to identify the relative location of the nerves along the SCM using real-time ultrasonography and to identify a safe location to prevent nerve injury during invasive procedures.

## 2. Methods

### 2.1. Subjects

This study was conducted from 2021 to 2022 on 52 neck samples from 26 healthy participants aged >19 years. Patients were excluded if they had a history of neck trauma or surgery, any neck deformity (torticollis or cervical dystonia), any medical conditions that could accompany peripheral polyneuropathy, or a history of central nervous system disease, neuromuscular junction disease, or myopathy. All volunteers were recruited from the community through discoveries at the Korea University Guro Hospital, and all participants provided written informed consent before the examination. This study was approved by the Institutional Review Board of the Korea University Guro Hospital.

Before ultrasonographic evaluation, baseline patient data were collected, including age, sex, height, weight, and total length of the SCM in the anterior, reference line (the midline between the anterior and posterior border of the SCM), and posterior border of the SCM.

### 2.2. Ultrasonographic examination

**Figure 1.**
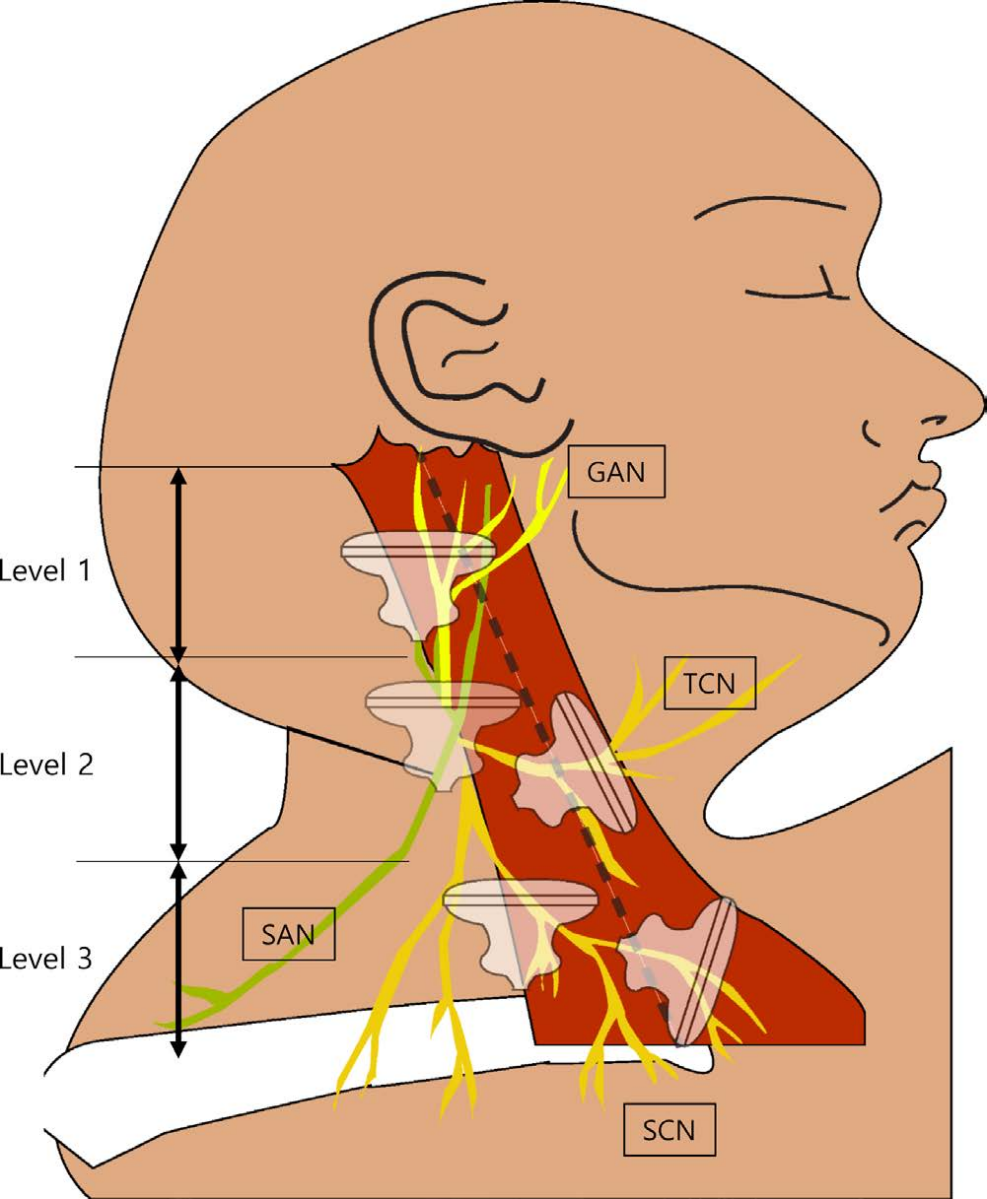
Schematic drawing shows the pathway of the great auricular (GAN), spinal accessory (SAN), transverse cervical (TCN), and supraclavicular (SCN) nerves which lie over or emerge underneath the SCM muscle. The dotted line indicates the reference line which is the midline between the anterior and posterior border of the SCM. All ultrasound probe marks on the figure represent the locations for visualizing nerves. SCM = sternocleidomastoid.

**Figure 2.**
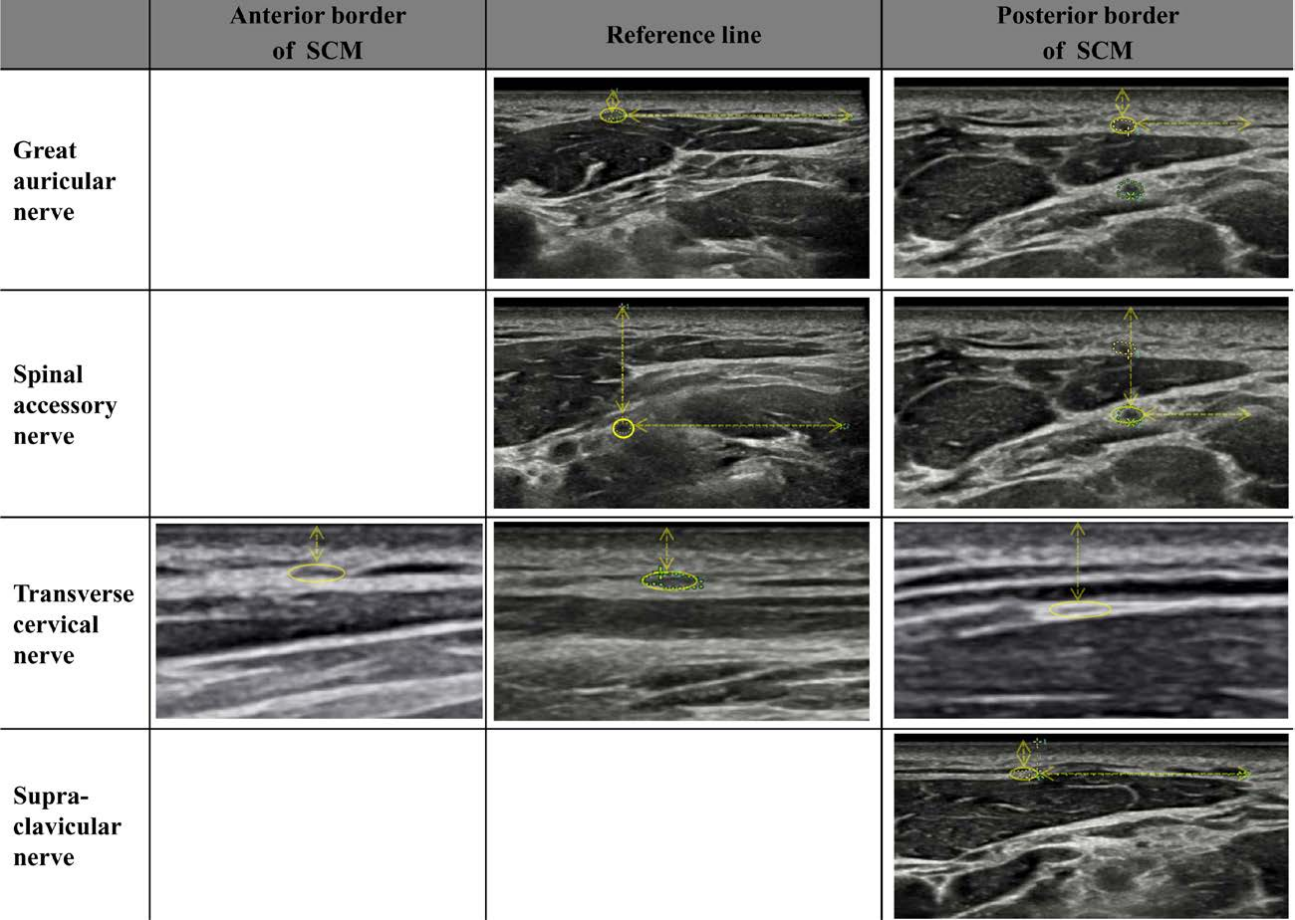
Ultrasonographic images of each nerve at the anterior border, reference line, and posterior border of SCM. Great auricular, spinal accessory, transverse cervical, and supraclavicular nerves were visualized with the transverse view of the path of each nerve. We measured cross-sectional areas, depth from the skin, and distance from the posterior border of the SCM. SCM = sternocleidomastoid.

One rehabilitation physician performed ultrasonography using an RS85 (Samsung Medison, Seoul, Korea) with a 3 to 16-MHz linear array transducer. The participants were scanned bilaterally in a standardized supine position, with the head turned 45° to the contralateral side. In half of the neck (level 2), the cervical plexus and TCN were located around the SCM muscle. First, we set Level 1 with the level for proper visualization of the GAN and SAN at the posterior border of the SCM, and Level 3 for the SCN. In the case of difficulty finding TCN, GAN was identified first and then scanned the caudal area relative to the GAN. We assumed TCN as the hypoechoic dot in this area which courses anteriorly and runs toward the midline of the ventral neck. With the sonographic transverse view for SCM, the GAN, SAN, and SCN were traced up to the level of contact with the anterior border of the SCM muscle (Fig. 1). We found the locations of the SAN and SCN which were superficial to the SCM, and SAN at the fascial layer underneath the SCM. The trapezoidal mode of the linear probe was used during the transverse scan of the anterior and posterior border of the SCM to identify the reference line at each level. With the longitudinal view for the SCM, TCN was scanned anteriorly from the posterior border of SCM. The following features of the nerves were recorded bilaterally: the vertical and horizontal positions of each nerve at the posterior border of the SCM and the cross-sectional area (CSA) and depth of each nerve from the skin at the reference line and anterior border of the SCM. Visualization of each nerve using ultrasonography at each corresponding level is presented in Figure 2. To distinguish the nerves from small vessels with a similar course, color Doppler served as an auxiliary method.

### 2.3. Statistical analysis

Statistical analysis was performed with the SPSS version 20.0 software (SPSS Inc., Chicago, IL). Normality of data was assessed using the Kolmogorov–Smirnov and Shapiro–Wilk tests. Quantitative data are presented as mean and standard deviation. Statistical significance was set at *P* < .05.

## 3. Results

**Figure 3.**
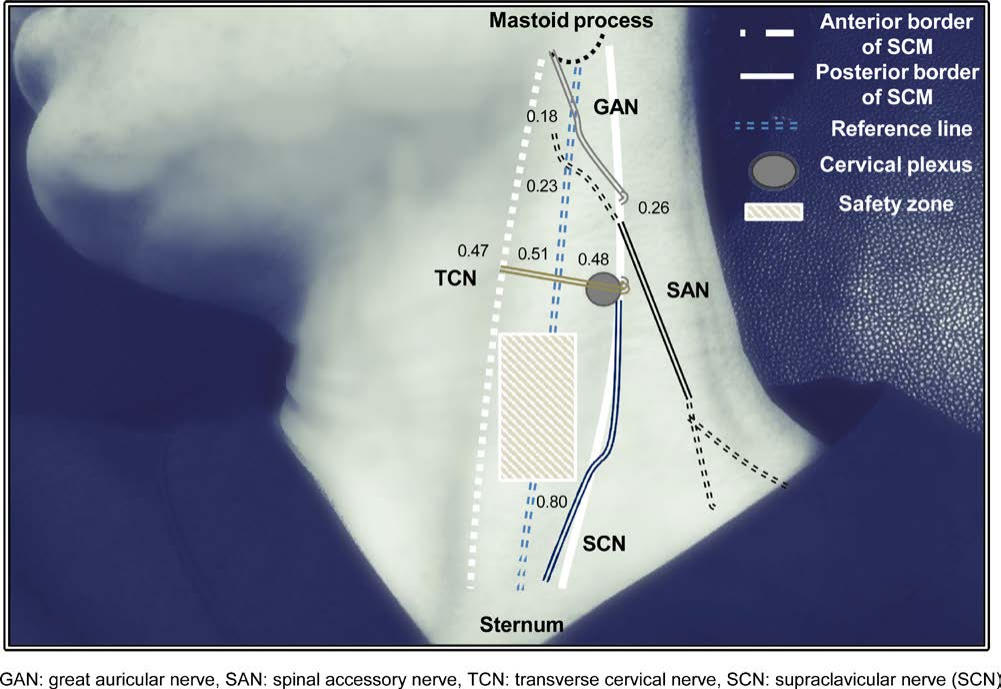
Anatomical relationship of each nerve and the sternocleidomastoid muscle. The reference line was defined as the line connecting the mastoid process and the sternal notch. Each number indicates the proportional location of the nerve in each line (the anterior border, reference line, and posterior border of the SCM). GAN = great auricular nerve, SAN = spinal accessory nerve; SCM = sternocleidomastoid, SCN = supraclavicular nerve, TCN = transverse cervical nerve.

A total of 52 samples from 26 healthy volunteers were included in the study. General baseline characteristics (sex, age, and body mass index) are presented in Table 1. We observed the cervical plexus and TCN in the mid-portion (approximately 48% of SCM). In the upper part, the GAN and SAN could be visualized in 26% of the SCM. Scanning, the distal part of the SCN was traced along the posterior border of the SCM and was well visualized at the 80% distal proportion in the SCM. The depth and horizontal distance from the posterior border of the SCM muscle are presented in Table 2. Along the posterior border, GAN, TCN, and SCN were the superficial structures which were 0.28 cm, 0.25 cm, and 0.27 cm away from the skin. The deepest structures were the cervical plexus at the middle level and the SAN at the proximal one-third level. The CSA and depth and relative locations of each nerve in the anterior border, reference line, and posterior border are shown in Table 3, and each suggested pathway is presented in Figure 3. Tracing along each nerve, the mean proportions of GAN, SAN, TCN, and SCN were 18%, 23%, and 51%, respectively, and the SCN was not visible at the level of the reference line. The mean TCN proportion was 47% at the anterior border of the SCM muscle. Only the SAN runs deep from the skin in the anterosuperior pathway, from the posterior border of the SCM. Other nerves were located at a mean depth of 0.18–0.27 cm which is relatively superficial compared to SAN. TCN and SCN showed relatively smaller CSA than GAN or SAN.

**Table 1:** Baseline characteristics of the study participants.

Variables	Mean ± standard deviation [range]
n	52
Mean age (yr)	36.8 ± 13.4 [25–67]
Mean height (m)	1.64 ± 0.08 [155–180]
Mean weight (kg)	62.7 ± 8.2 [44–80]
Mean BMI (kg/m^2^)	23.0 ± 3.2 [18.3–27.7]
Total length of SCM (cm)	14.9 ± 1.6 [12–18.5]

Total length of SCM refers to the distance from mastoid process to sternoclavicular notch.

BMI = body mass index, SCM = sternocleidomastoid.

**Table 2:** Ultrasonographic characteristics of the study participants.

	Distance from MP			Relative location in SCM	
Level 1 (proximal 1/3)	3.88 ± 0.86			26.24 ± 5.98 %	
		GAN	Distance from SCM posterior border [medial (−), lateral (+)]		−1.64 ± 0.48
			Depth		0.28 ± 0.08
		SAN	Distance from SCM posterior border [medial (−), lateral(+)]		−1.27 ± 0.44
			Depth		0.61 ± 0.10
Level 2 (middle 1/3)	7.26 ± 1.18			48.67 ± 5.61 %	
		TCN	Depth		
		Cervical plexus	Distance from SCM posterior border [medial (−), lateral(+)]		0.25 ± 0.09
			Depth		−1.35 ± 0.65
					0.80 ± 0.17
Level 3 (distal 1/3)	11.97 ± 1.75			80.34 ± 7.70 %	
		Supraclavicular nerve	Distance from SCM posterior border [medial (−), lateral(+)]		−0.95 ± 0.66
			Depth		0.27 ± 0.07

Vertical and horizontal distance and depth of the location of nerves around the SCM using landmarks. (MP: mastoid process, anterior border: attachment site of SCM on sternal notch, posterior border: posterior margin attachment site of SCM on clavicle.)

GAN = great auricular nerve, SAN = spinal accessory nerve, SCN = supraclavicular nerve (SCN), TCN = transverse cervical nerve

**Table 3:** Characteristics and relative position of each nerve around sternocleidomastoid muscle.

Nerves	Anterior border of SCM	Reference line	Posterior border of SCM
Greater auricular nerve			
CSA (mm^2^)	Nonvisible	0.51 ± 0.02	0.63 ± 0.05
Depth (cm)	Nonvisible	0.28 ± 0.12	0.28 ± 0.08
Proportion (mean [range])	Nonvisible	0.18 [0.10–0.24]	0.26 [0.19–0.36]
Spinal accessory nerve			
CSA (mm^2^)	Nonvisible	0.36 ± 0.02	0.71 ± 0.07
Depth (cm)	Nonvisible	0.78 ± 0.35	0.61 ± 0.10
Proportion (%, Mean[range])	Nonvisible	0.23 [0.19–0.28]	0.26 [0.19–0.36]
Transverse cervical nerve			
CSA (mm^2^)	0.31 ± 0.21	0.36 ± 0.15	0.33 ± 0.07
Depth (cm)	0.24 ± 0.12	0.22 ± 0.08	0.25 ± 0.09
Proportion (%, mean [range])	0.47 [0.43–0.52]	0.51 [0.46–0.53]	0.48 [0.42–0.57]
Supraclavicular nerve			
CSA (mm^2^)	Nonvisible	Nonvisible	0.29 ± 0.07
Depth (cm)	Nonvisible	Nonvisible	0.27 ± 0.07
Proportion (%, Mean [range]	Nonvisible	Nonvisible	0.80 [0.70–0.95]

CSA = cross-sectional area, SCM = sternocleidomastoid.

## 4. Discussion

According to our study results, it is recommended to perform an invasive procedure in the lower half of the SCM muscle, which refers to 50 to 80% proportions in our study. Many ultrasonographic studies have targeted the cervical plexus for the nerve block to provide adequate anesthesia during neck surgery.^[9,10]^ In addition, dorsal scapular nerve injury is usually reported with neck surgery involving the posterior triangle.^[4]^ However, few studies have focused on the branches of the cervical plexus and the nerves traveling along the SCM.^[11]^

Previous cadaveric studies reported the SAN was identified 8.2 + 1.01 cm cranial to the clavicle on the posterior border of the SCM.^[12]^ Although our SAN findings were anterior-inferiorly located, these findings are similar to previous findings.

SCN has several branches which include lateral, intermediate, and medial branches. However, in this study, we aimed at the medial branch which is estimated to locate around the SCM. Nathe et al confirmed the location of the SCN in the clavicle, there was no supraclavicular nerve within 2.7 cm of the sternoclavicular joint.^[13]^ Inferred from this cadaver study, the anteroinferior portion of SCM is the safe area. This data is compatible with our study in that the SCN was found only around the posterior border of the SCM at the 80% proportion of total SCM length.

Lefkowitz reported that GAN at its most superficial location was found at the proximal one-third distance within SCM.^[14]^ In this study, we visualized GAN at the mean proportion of 26%, and this indicates a similar aspect to the previous study.

In a previous ultrasonographic study, Drlicek G et al reported that TCN located 10 to 11 mm caudally from the GAN at the posterior border of the SCM muscle.^[15]^ However, we found out TCN was 3.3 mm (22% of total SCM length) away from GAN. In this study, we visualized GAN at a slightly cranial level to distinguish it from other possible structures such as SAN. The different measurement methods might be the possible reason which caused the difference. Still, this difference doesn’t change the fact that we have to be cautious when approaching the proximal 50% portion of SCM.

Some studies have used ultrasound to block GAN,^[16]^ and there are no available ultrasonographic data for SCN for comparison.

During invasive procedures, such as trigger point injection or botulinum toxin injection, there is no consensus on the approach of the SCM muscle. Previous botulism injection studies^[6]^ suggest that the proximal portion should be selected because minimizing botulinum toxin diffusion to the swallowing area is important. Kim et al^[17]^ reported that targeting the exact SCM muscle is better to target the mid-portion of the SCM muscle. However, they did not focus on the possibility of potential nerve injury risk. Although real-time imaging is the best way to prevent possible complications during invasive procedures, it is not always possible, depending on the clinical setting. Considering our study results, it is not recommended to approach the proximal and middle parts of the SCM without imaging devices. Moreover, the relatively small CSA of the TCN and SCN indicates a low possibility of nerve injury. This finding could also lead to the conclusion that the lower third is safer than the upper half of the SCM.

This study had some limitations. First, the sample size is relatively small. Therefore, a large-sample study should be conducted in the future. In addition, our patient population had a low body mass index, which might have affected the depth or location of each nerve. Third, ultrasonography and analysis were performed by a single physician. Intra- and inter-rater reliabilities were not evaluated. Fourth, we only analyzed the most anteriorly situated branches which are the medial branch of the SCN and the anterior portion of the GAN.

In conclusion, the precise location of the nerves and their relationship with the SCM should be considered during invasive procedures. It is safe to perform invasive procedures in the distal 50 to 80% of SCM cases. For the proximal site of the SCM, the use of additional imaging devices such as ultrasonography is recommended.

## Acknowledgments

This research was supported by the Basic Science Research Program through the National Research Foundation of Korea (NRF) funded by the Ministry of Science, ICT & Future Planning (NRF-2020R1A2C1009024).

## Author contributions

**Conceptualization:** Byung Heon Kang, So Hyun Park, Seok Kang, Joon Shik Yoon.

**Data curation:** Byung Heon Kang, So Hyun Park.

**Formal analysis:** Byung Heon Kang.

**Funding acquisition:** Joon Shik Yoon.

**Investigation:** So Hyun Park, Joon Shik Yoon.

**Methodology:** Byung Heon Kang.

**Resources:** Byung Heon Kang.

**Supervision:** Seok Kang, Joon Shik Yoon.

**Validation:** Byung Heon Kang, Seok Kang.

**Visualization:** Byung Heon Kang.

**Writing – original draft:** Byung Heon Kang, So Hyun Park.
